# Psychometric Assessment of the European Health Literacy Survey Questionnaire (HLS-EU-Q16) for Arabic/French-Speaking Migrants in Southern Europe

**DOI:** 10.3390/ijerph17218181

**Published:** 2020-11-05

**Authors:** Pilar Bas-Sarmiento, Miriam Poza-Méndez, Martina Fernández-Gutiérrez, Juan Luis González-Caballero, María Falcón Romero

**Affiliations:** 1 Department of Nursing and Physiotherapy, University Institute of Research in Social Sustainable Development (INDESS), University of Cadiz, 11009 Cadiz, Spain; pilar.bas@uca.es; 2 Department of Nursing and Physiotherapy, Faculty of Nursing, University of Cadiz, 11207 Cadiz, Spain; miriam_alge@hotmail.com; 3 Department of Statistics and Operational Research, Faculty of Medicine, University of Cadiz, 11003 Cadiz, Spain; juanluis.gonzalez@uca.es; 4 Department of Socio-Sanitary Sciences, University of Murcia, 30100 Murcia, Spain; falcon@um.es

**Keywords:** health literacy, migrants, psychometrics, reliability and validity, surveys and questionnaires

## Abstract

Improving health literacy (HL) is critical for addressing health inequalities. Low literacy rates are believed to be more prevalent in ethnic minorities, which may have an impact on people’s health. For measures to be implemented in this regard, HL must be evaluated to obtain specific indicators. Our aim, therefore, was to develop a version of the European Health Literacy Survey Questionnaire (HLS-EU-Q16), which is recommended to be used with vulnerable populations, suited to Arabic/French-speaking migrants who reside in south-eastern Spain, and to explore its psychometric properties for assessing health literacy in this population. A cross-sectional survey was carried out in a convenient sample of 205 migrants. The structural validity was calculated by a confirmatory factorial analysis (CFA), which suggested appropriate adjustment indicators, and which indicated that the three-dimensional model is adequately adjusted to the data obtained in the study. The coefficient omega showed high internal consistency in the three HL dimensions (health care, disease prevention, and health promotion). Concurrent validity presented a significant correlation with the Newest Vital Sign test (r = 0.390; *p* < 0.001). The multigroup CFA showed that the heterogeneity of the sample used was not a problem for establishing the structural validity of the scale. The Arabic/French version showed good construct validity.

## 1. Introduction

Since the term Health literacy (HL) emerged in the 1970s [1], the meaning and conceptual model of the term have evolved from basic reading and writing skills in the health care context to a more complex and multidimensional construct. After a systematic literature review of definitions and models to identify the central dimensions of health literacy, the European Health Literacy Survey Project (HLS-EU) was seen to provide a comprehensive definition: “The people’s knowledge, motivation and competences to access, understand, appraise and apply health information in order to make judgments and take decisions in everyday life concerning health care, disease prevention and health promotion to maintain or improve quality of life throughout the course of life” [2].

In accordance with this definition, a conceptual model was developed that includes the main dimensions and factors that have an impact on HL, as well as the pathways linking HL with health outcomes. This model, which was used in our study, combines four key processes that refer to health information processing (competences to access, understand, assess, and apply health information) and three health domains (health care, disease prevention, and health promotion). The model comprises a matrix with 12 cells, and was the starting point for developing a questionnaire to measure health literacy: The European Health Literacy Survey Questionnaire (HLS-EU-Q47) [3].

The HLS-EU-Q47 has been widely used in many different countries and is available in a range of languages [3,4,5]. This instrument addresses self-report difficulties in tasks concerning decision-making in health care (HC), disease prevention (DP), and health promotion (HP). Each sub-dimension of the comprehensive health literacy concept is represented by a specific score: a general-HL score comprising all items, and three specific sub-dimension scores covering health care (HC-HL), disease prevention (DP-HL), and health promotion (HP-HL) [6].

A short version of the tool comprising 16 of the 47 original items is frequently used in population studies since it can be completed quickly. The HLS-EU-Q16 is easy to analyze, has a high correlation with the original version (r = 0.82) [6] and is recommended for vulnerable populations [7]. However, although it has been translated into Dutch [7], Swedish [8], German [9], Norwegian [10], Spanish [11], Italian [12], Greek [9], Czech [13], Hebrew [14], Arabic [15,16], French [17], Icelandic [18] and several Asian languages [5,19], there is little information about its psychometric characteristics. Furthermore, most of these versions have been developed in the general population, except in the case of the Arabic version [15] that has been used in the immigrant refugee population in Sweden. Therefore, studies that evaluate its use in different migrant contexts and populations are necessary.

There is strong evidence of limited HL throughout the world [4,5,9,20,21]. People with limited HL are more likely to have difficulty with processing health information to take care of their own health [2], and are associated with poor health outcomes [19,21,22] due to medication errors [23] and/or poor understanding of diseases and treatments. HL plays a crucial role in patients with chronic diseases [24] and in especially vulnerable groups, such as migrants and refugees [10,15,25,26,27,28,29].

By contrast, good HL promotes better self-care, better health outcomes, and lower health costs [30]. Similarly, health literacy is as important for the prevention of communicable diseases as it is for non-communicable diseases. The COVID-19 pandemic has also been an “infodemic”, since poor health literacy among a given population has been seen to be a public health problem globally [31]. Health literacy might help people to understand the reasons behind any recommendations and to reflect on the possible outcomes of their actions [32]. Thus, health literacy is increasingly recognized as an important health determinant [32,33], and several studies have confirmed the existence of a social HL gradient that results in health inequality for the most disadvantaged groups [9], as is the case with migrant populations [34].

In the European Region, almost 10% of the population are international migrants and, in Spain, the figure is around 13% [35]. In recent years, the proportion has slightly increased in south-eastern Spain with an immigrant share of 13.01%, many of them from North Africa and sub-Saharan countries [36]. Indeed, migratory flows from Africa have remained constant and the region continuously receives small vessels crammed with immigrants, including 1192 in 2019 [37].

There has been a call upon member states of the European Union to promote the health of refugees and migrants through sensitive health policies and health care provision that ensures equitable access to health information and care [38]. However, if this is to be achieved, it is of paramount importance to properly measure HL in migrants, so that their specific needs can be addressed, and the challenges faced by health systems to provide care for these multilingual and multicultural communities be overcome. Assessing health literacy in this population may identify indicators for tailoring health policies, implementing effective interventions and generally improving the well-being of this population in the host countries.

As has been seen, the HLS-EU-Q16 may offer help in this respect as long as it is adapted to the cross-cultural sensitivity of the population in question. As the majority of our target population speaks Arabic and/or French languages, the purpose of this study was to develop an adaptation of the HLS-EU-Q16 for Arabic/French-speaking migrants who currently reside in south-eastern Spain, and to explore its psychometric properties for assessing health literacy in this migrant population.

## 2. Materials and Methods

### 2.1. Study Design and Setting

A cross-sectional survey was carried out from May to October 2017 in the south of Spain (Campo de Gibraltar), a Mediterranean coastal area in the province of Cadiz, which acts as a gateway to Europe for many immigrants from Africa since it is the southern-most part of the Iberian Peninsula and therefore nearest to the Moroccan coast. At the time of data collection, Cadiz province had a total of 40,938 immigrants, of whom 20,036 (48.94%) lived in the Campo de Gibraltar. The figure of 7440 were from Africa and, of these, 6935 were from Morocco (93.21%) [39].

### 2.2. Instruments

The HLS-EU-Q16 contains 16 items addressing self-reported difficulties in accessing, understanding, appraising and applying information to tasks related with making decisions in health care, disease prevention, and health promotion. The HLS-EU-Q16 was obtained from the HLS-EU-Q47 version with items based on Rasch Analysis [6]. Each item was rated on a four-point Likert scale (very difficult, difficult, easy, and very easy) and a “don’t know/no answer”. Following the authors’ instructions, when scoring the HLS-EU-Q16, the categories “very difficult” and “difficult” are scored as 0, and the categories “easy” and “very easy” are scored as 1. Scale values are calculated as simple summed scores only for respondents who answered at least 14 items. Scoring varies between 0 and 16, establishing three levels of HL: inadequate (0–8), problematic (9–12), and sufficient (13–16) [6,40]. The 16 items in the questionnaire represent 11 out of the 12 cells in the HL-EU matrix, and, similarly to the HLS-EU-Q47, the Q16 shows a partial score for the domain of health care, disease prevention, and health promotion. Correlations with the long version of the form (HLS-EU-Q47) were very high (r = 0.82 for the total sample) and varied between 0.73 and 0.88 for different countries [6].

The Newest Vital Sign test (NVS) [41] was used to examine concurrent validity. The NVS consists of an ice cream nutrition label, with seven associated questions that measure literacy and numeracy. It produces a final score of functional HL ranging from 0 to 6. Scores were classified as high probability of marginal/insufficient HL, possibility of marginal/inadequate HL, and adequate HL. The NVS is a valid and reliable screening tool available in English and Spanish that identifies patients at risk for low health literacy [41], and in the European Health Literacy Survey, it was correlated with both the HLS-EU-Q47 (r = 0.266) and the HLS-EU-Q16 (r = 0.231) [6].

### 2.3. Translation

The HLS-EU-Q16 was translated into Arabic and French, trying to ensure that the resulting questionnaires maintained the semantic, idiomatic, conceptual, and experiential equivalence of the original. Thus, the questionnaire was translated and adapted following the consensus on reported methodology [42,43,44,45], which consists of five phases:

Direct translation: The Spanish version of the questionnaire was provided to two Arabic translators whose mother tongue was Arabic. One of them, a medical translation expert (with wide experience in translating health education-related resources for this specific population), was informed of the objective of the study, whereas the other was not. The same was done for the French version. In this phase, two versions of the questionnaire were obtained in French and two in Arabic.

Translation synthesis: Simultaneous discussion among the translators led to a consensus being reached, generating the first versions of the questionnaire in Arabic and French.

Back translation: Two bilingual translators whose mother tongue was Spanish and who had not participated in the previous stages, worked independently, without seeing the original version of the questionnaire and were unaware of the objectives of the study. The retro-translation gave rise to no major semantic or conceptual differences.

Consolidation by a committee of experts: A consensus was reached between the participants of the previous phases, and a final document that contained the translations of both languages was obtained constituting the Arabic/French final version.

Pre-Test: As a pre-test exercise, the questionnaire was given to 15 people and, in light of their answers, it was seen that no changes needed to be made.

### 2.4. Sample/Participants

Convenient sampling was used to recruit participants (*N* = 205), who were enlisted from key informants and from those answering an advertisement distributed by migration-related associations (Andalusian Acoge, Spanish Red Cross, White Cross Foundation, Intercultural Association ESMAR, Prolibertas Foundation, Coexistence and the Social Cohesion Foundation (CEPAIM is its Spanish acronym), and the Association of Women in Active Job Search (AMBAE)). The inclusion criteria were being an Arabic-French-speaking migrant, resident in Spain and aged at least 16 years. The exclusion criteria were not speaking Arabic and/or French languages. For the sample size, we chose the 10:1 participant rule: item ratio to determine a sufficient sample size [46,47]. As the HLS-EU-Q16 consists of 16 items, the number of participants was set at 160, which satisfied the requirements of confirmatory factorial analyses (CFA). However, as larger samples provide accurate estimates of the factorial loads of each item and establish more stable factors [46], an effort was made to maximize the sample size and thus recruit a higher number of participants than at first proposed.

### 2.5. Data Collection

The survey including the NVS, the HLS-EU-Q16, and several additional sociodemographic questions were conducted by trained cultural mediators individually or in small groups and completed by each participant without help, except when the mediators or key informants/helpers read the questionnaires to participants who were not literate. Each questionnaire took about three to five minutes.

### 2.6. Data Analysis

To determine the normality of the sample distribution for each of the studied numeric variables, the Kolmogorov-Smirnov test was used. Using descriptive statistics, the sociodemographic variables were summarized, as well as the distribution of the total scores and each item of the scale. Meanwhile, the internal consistency, and construct validity (CFA, hypothesis-testing and criterion-related validity by concurrent validity) were assessed.

McDonald’s omega was calculated to evaluate the internal consistency of the measurement [48,49]. Unlike the alpha coefficient, the omega coefficient works with factorial weights [50], which are the weighted sum of the standardized variables, a transformation that makes the calculations more stable [51] and reflects the true level of internal consistency and does not depend on the number of items. For the reliability of the omega coefficient to be considered sufficient, a value of 0.70–0.90 must be attained [48].

To establish structural validity, a CFA was conducted consisting of the three domains of health: Health care, disease prevention, and health promotion. Tetrachoric correlations were used between the items as input for the data analysis and the weighted least square (WLSMV) method to obtain parameter estimates. To evaluate the fit of the data to the proposed model in each case, the fit rates suggested by Kline [52] were used: a non-significant chi-square test; a Comparative Fit Index (CFI) greater than 0.90; a Tucker-Lewis Index (TLI) greater than 0.90; Root Mean Square Error Approximation (RMSEA) less than 0.08 and Standardized Root Mean Square Residual (SRMS) less than 0.06.

Invariance of the obtained dimensions [53,54] across population groups was evaluated using multi-group modeling. As the items are binary, only the configural and scalar invariance were considered [55]. To evaluate the changes in the multi-group models, we used the standard changes in the absolute values of the fit indices [56], that is |ΔCFI| ≤ 0.010, |ΔTLI| ≤ 0.010, |ΔRMSEA| ≤ 0.010.

To determine the hypothesis testing (known-groups validity), the instrument was evaluated in groups that were presumed to differ in an attribute measured by the instrument due to a known characteristic. In the case of HL, the demographic attribute determines a differential response between education and HL level [9,19,57], and between income and HL level [9,17]. To compare the groups, we used a non-parametric test for K-independent samples (Kruskal-Wallis test).

Concurrent validity: The correlation between HL measured by the HLS-EU-Q16 and by the NVS was examined by Spearman’s correlation coefficient. A value equal to or higher than 0.80 indicated that the instruments were psychometrically equal. If the purpose is to show that a new scale is more valid and better than a reference instrument, the ideal would be to obtain correlations of between 0.3 and 0.7, which would indicate that the two instruments are different despite measuring the same attribute [42,58].

Analyses were performed using the statistical package SPSS, version 22 (2015) (IBM, Chicago, IL, USA), and Mplus, version 8.4, for CFA (Muthén & Muthén, Los Angeles, CA, USA) [59]. Statistical significance was determined with a value of *p* < 0.05.

### 2.7. Ethical Considerations

The aim of the study and the anonymity of participants, as well as the voluntary character of participation, were all explained before the participants started answering the questionnaire and their informed consent was obtained. The participants were also informed that the data obtained would be used for research purposes only.

## 3. Results

### 3.1. Translation

The final adaptation of the questionnaire, available in Supplementary Materials (Table S1) was semantically, conceptually, and experientially equivalent to the original version.

### 3.2. Sample Characteristics

The sample was composed of 205 participants: 110 of Moroccan origin; 56 of sub-Saharan origin; and 39 from other Arabic-French-speaking African countries. All participants completed the total number of items in the questionnaire. The mean age was 33.92 years (SD = 11.09 years). Socio-demographic characteristics of the participants are detailed in Table 1.

### 3.3. Construct Validity

#### 3.3.1. Structural Validity

**Figure 1:**
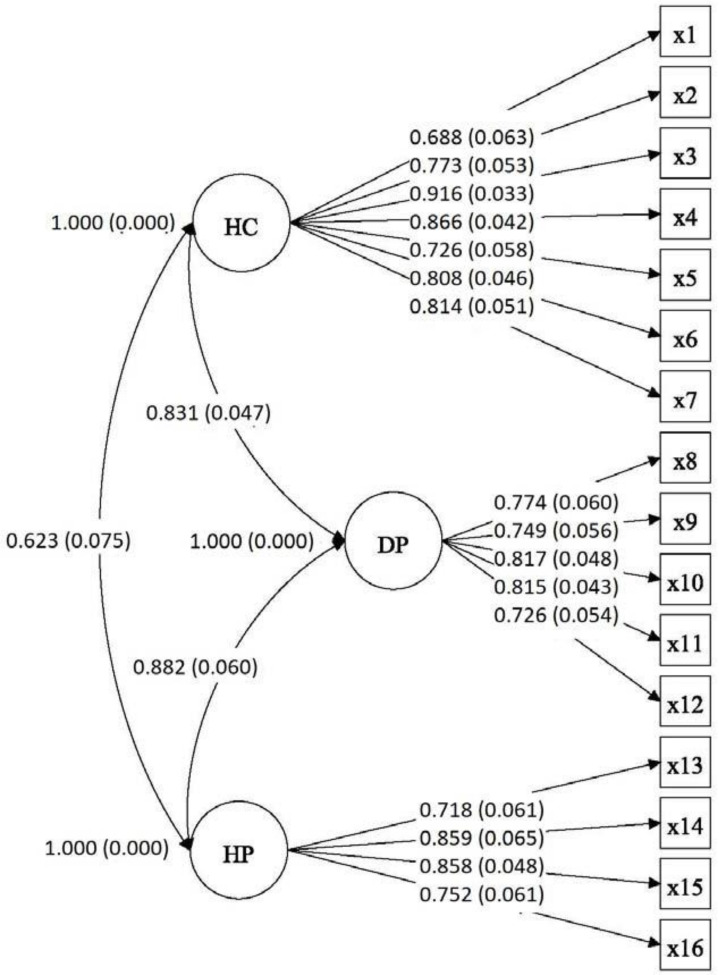
CFA model. Note: HC: Health Care, DP: Disease Prevention; HP: Health Promotion and x is the item.

The results of the CFA performed to evaluate the three-dimensional model of the HLS-EU-Q16 are presented in Figure 1. The adjusted rate obtained was as follows: χ^2^ = 174.344, df = 101, *p* < 0.000; CFI = 0.963; TLI = 0.953; RMSEA = 0.06 (90% CI = 0.044–0.074); SRMS = 0.09, suggesting that the three-dimensional model (health care, disease prevention, and health promotion) was adequately fitted to the data obtained in the study. The correlations between the three factors indicate that, although there are three dimensions, they are closely related, which means that a common dimension (Health Literacy) can be established.

The coefficient omega was calculated for the subdimensions health care (HC-HL), disease prevention (DP-HL), and health promotion (HP-HL); the values obtained were 0.874, 0.833, and 0.798, respectively. The square multiple correlation variability ranged from 0.47 to 0.84, with a mean of 0. 629.

The assessment of MI in the CFA model among the three population groups, Moroccans (MA), sub-Saharans (SS) and other nationalities (ON), can be seen in Table 2. The adjustment of model parameters is mostly appropriate, and the changes in the absolute values of the fit indices are within those proposed by Chen [56].

#### 3.3.2. Hypotheses-Testing (Known Groups Validity)

The literature coincides in stating that education level and perceived financial situation are related to the level of HL [9,17,19,57]. However, in this case, we observed no significant differences between the HL scores and education level (χ^2^ = 7.428; df = 5; *p* = 0.191), so that this hypothesis cannot be confirmed. However, there were significant differences between HL level and monthly income (χ^2^ = 18.64; *p* = 0.001), showing that, in this case, the validity of known groups was confirmed.

#### 3.3.3. Criterion Validity

The criterion validity was measured using the concurrent validity: Spearman’s correlation coefficient showed a significant relation between HLS-EU-Q16 and NVS (r = 0.390; *p* = 0.000). As Spearman’s correlation coefficient was set at 0.3–0.7, the two questionnaires seemed to differ despite measuring the same attribute.

### 3.4. Distribution of Scores

The average HL value obtained in the sample using the HLS-EU-Q16 questionnaire was 9.65 (SD = 4.51), corresponding to the problematic HL level (Table 3). The range of scores observed was 0 to 16 points, concurring with the minimum and maximum scores that the scale could theoretically yield. Of the whole sample, 39% of participants showed inadequate HL, 32.2% sufficient and 28.8% problematic.

The average scores in females were higher than in males to a statistically significant extent (t = −2.011; *p* = 0.046). A significant degree of correlation was also observed between age and HL (r = 0.250; *p* = 0.000). Table 4 shows the scores of the different age groups.

## 4. Discussion

In this study, an Arabic/French version of the European questionnaire HLS-EU-Q16 was prepared, following transcultural adaptation. The dimensions proposed by Flaherty et al. [60] were respected which ensured the linguistic and cultural equivalence of the new version and maintained the psychometric characteristics of the original instrument. Using this method ensured the high quality of the translation process [44,45,61].

The CFA results supported the construct validity of the HLS-EU-Q16. The structural validity yielded acceptable adjustment indices, endorsing the described factorial structure. The three-dimensional model (health care, disease prevention, and health promotion) was adequately adjusted, as in studies carried out in other contexts [5,62].

The HLS-EU-Q16 questionnaire shows evidence of adequate internal consistency, as demonstrated by the omega coefficient values, in consonance with other studies carried out using Cronbach’s α coefficient: Spain = 0.982 [11]; France = 0.81 [17]; Israel = 0.928 [14]; Turkey = 0.89 [62]; Norway = 0.91 [10]; Italy = 0.799 [12], and Iceland = 0.88 [18].

In the health care (HC-HL) and disease prevention (DP-HL) domains, satisfactory results were obtained in terms of the omega coefficient (0.874 and 0.833, respectively), which are similar to the values obtained in other studies [5,6,16]. In the health promotion domain (HP-HL), the omega coefficient was slightly low (0.798), but this value cannot be considered poor or unacceptable. In the European Health Literacy Project [3], the results obtained for HP-HL were also lower compared with the other domains: 0.739 in the European and 0.660 in the Spanish sample [6].

From the assessment of MI in the CFA model, it can be affirmed that the heterogeneity of nationalities in the sample used in the study was not a problem for establishing the structural validity of the scale.

In the validity of known groups, it was not possible to establish a significant relationship between HL and education level, which agrees with Rouquette et al. [17]. This may be attributed to the sample, being obtained by a non-probabilistic sampling of convenience, and the fact that most people in the sample had attended secondary education. It would be interesting for future research to take a more heterogeneous sample with respect to this variable, as the evidence shows a positive correlation between formal education and HL [14,17,21,60]. However, it was possible to establish a significant relationship between HL level and financial situation, which agrees with previous studies [9,17].

With respect to the concurrent validity, the results showed a significant correlation between both measuring instruments (HLS-EU-Q16 and NVS), again similar to the results obtained by others [5,6,41,58]. Thus, the HLS-EU-Q16 could be used to compare the level of HL and its associated factors between countries that have significant cultural variations [5].

The HL of the participants corresponded to a problematic average HL level of 9.65 (SD = 4.51), in line with previously analyzed data in the international context. A decade ago, the U.S. Department of Health and Human Resources [63] launched the National Action Plan for the improvement of HL, finding that limited HL was a major public health problem [64]. At European level, the results of the HLS-EU project showed that limited HL (sum of problematic and inadequate HL) is not a concern limited only to minority groups: over 12% of respondents (*n* = 8000) presented an inadequate HL level, and more than 35%, a problematic level [9]. Most recent studies found similar results, with 30.94% of the population having a problematic level of HL in Denmark [65], and 27.5% an inadequate HL in Iceland [18]. Studies carried out in immigrant populations are scarce [66]. The most outstanding initiatives come from the United States, where studies focused on Latin and Asian populations although they did not measure health literacy in all its dimensions, and did not take into account the context in which the individual is immersed [25]. The study of Geltman et al. [26] with Somali refugees (*n* = 432) in Massachusetts showed that 74% had low functional HL, while Gele et al. [10] obtained a similar percentage of low HL (71.4%), among Somali refugee women. Sentell and Braun [28] identified a low HL level in 44.9% of a sample made up of Chinese, Latinos, Koreans, and Vietnamese in California. Meanwhile, in Sweden [15], almost 80% of refugees had a limited HL (both inadequate and problematic). Likewise, Fernández-Gutiérrez et al. [67] reported that 70.41% had inadequate and problematic HL in a study based on 93 participants of 17 distinct nationalities in Spain. In the present work, 67.8% of participants were found to have a limited level of HL (inadequate and problematic).

With regard to gender, the scores of women were higher than those of men and the average of the total sample. According to this variable, there were significant differences in the HP-HL (Health Promotion) domain. Similar results were found in a meta-analysis carried out in the United States [68] and in Turkey [62]. In the studies of Almaleh et al. [16], conducted with clinic outpatients in Egypt, the largest factor shaping health literacy was gender (men had a lower health literacy level than women). However, in other surveys, no significant gender impact on the level of health literacy was found [12,18].

Nevertheless, there are examples in the scientific literature that provide evidence for men having a higher score than women [69]. In a review on the use of HLS-EU-Q16, Niedorys et al. [70] argued that a low level of health literacy was more common in African women and was also associated with the material status of the surveyed people. In Ghana, according to Amoah et al. [71], this situation was caused by the poor economic situation of the state, limited educational opportunities and a patriarchal social structure that places the needs of men over the needs of women. Similar results were obtained by Levin Zamir in Israel [14]. In our case, although the sample population probably maintains a patriarchal structure, and the percentage of women without formal education is higher in general, most of our participants (both women and men) had studied in secondary schools, which may explain the results obtained.

Studies reporting an association between health literacy and age showed differences between countries in the European Health Literacy Survey, HL falling with age in all countries except for the Netherlands [6], and a recently published study from Japan showed an increase in HL with age [4]. In the present study, HL scoring had a significant positive correlation with age, although if the age variable is broken down into categories, as in Table 4, it is seen that the older the participant, the higher the score obtained in the questionnaire, but only up to the age of 65. These results may be explained by reference to previous studies that found that cognitive deterioration with age can negatively affect the level of HL [72,73].

### Limitations, Strengths, and Practical Implications

The current study has a number of limitations. Firstly, it uses a non-probabilistic sampling method of convenience. A convenience sample can lead to the over-representation of particular groups within the sample. Since the sampling frame is not known, and the sample is not chosen randomly, the inherent bias in convenience sampling means that the sample is unlikely to be representative of the population being studied. On the other hand, despite the fact that the sample is heterogeneous, especially in the sub-Saharan group, evaluation of the invariance enabled the coherence of the sample to be demonstrated for this questionnaire.

Secondly, although the Arabic/French adaptation was based on the Spanish version, which may be considered as a limitation, later comparisons with the original source did not detect semantic differences.

Similarly, as HL is context-dependent, meaning that it may vary in different environments, caution is needed in any generalization of our results. Finally, it is necessary to point out that additional studies are needed to evaluate the psychometric properties of the Arabic/French version, and that it was not possible to evaluate the stability of the responses over time (i.e., test-retest reliability).

Nonetheless, in health and epidemiological research, the tool can be used by different health professionals to assess HL in migrant populations, and to obtain information that will help guide decision-makers toward maintaining and improving the health of this population by, for example, adapting health education to the level of HL indicated by the questionnaire results. The tool can also be used to measure the impact of interventions that are carried out to improve the level of HL in the population.

The main strength of this study is that it shows that it is possible to develop, implement and validate a culturally appropriate HL questionnaire that is responsive to the needs of a migrant population. This population group has, in general, been poorly represented in research on health literacy assessment, reflecting a significant research gap.

Improving health literacy implies establishing effective health policies and making efforts to promote health. For that purpose, instruments are needed to assess the deficiencies and potentials in specific contexts and populations. The developed version will facilitate this work and may also be useful for evaluating health literacy in other contexts and populations.

## 5. Conclusions

As confirmed by the data obtained in this study, the cross-cultural adaptation of HLS-EU-Q16, with its internal consistency and construct validity, can be used to evaluate HL in immigrant populations in the same way as the original version.

This study is the first to investigate the psychometric properties of the HLS-EU-Q16 questionnaire in a migrant African population in south-eastern Europe which could also be used in other contexts to obtain data on the health literacy of vulnerable populations. However, new validation studies are needed to consolidate the results obtained, both with respect to the psychometric properties and to provide comparative data.

## Figures and Tables

**Table 1:** Socio-demographic characteristics of the participants.

Socio-Demographic Characteristics	*n*	%
Sex		
Male	83	40.5
Female	122	59.5
Age		
Under 35 Years	121	59
From 36 to 50 Years	67	32.7
From 51 to 65 Years	12	5.9
Older than 65 Years	3	1.5
Missing values	2	0.9
Country/Region of Origin (grouped)		
Moroccan	110	53.7
Sub-Saharan	56	27.3
Other	39	19.0
Time in Spain		
Less than a Year	39	19.0
From 1 to 3 Years	34	16.6
More than 3 Years	131	63.9
Missing Values	1	0.5
Education Level		
No Formal Education	14	6.8
Primary	15	7.3
Secondary	128	62.4
University Studies	18	8.8
Does not know/does not answer	30	14.6
Status of Employment		
Working	51	24.9
Unemployed/Inactive	92	44.9
Student	11	5.4
Retired/Permanent Disability	6	2.9
Housewife	23	11.2
Does not know/does not answer	22	10.7
Difficulties to pay Bills at the End of a Month		
Yes	115	56.1
No	40	19.5
Does not know/does not answer	50	24.4
Household’s Net Income per Month		
Less than EUR 800	76	37.1
Between EUR 800 and 1350	24	11.7
Between EUR 1350 and 2400	7	3.4
More than EUR 2400	1	0.5
Does not know/does not answer	97	47.3

**Table 2:** Measurement of invariance in the CFA model.

Comparisons	WLSMV χ^2^ (df)	*p*	CFI	TLI	RMSEA	Δχ^2^ (df)	*p*	ΔCFI	ΔTLI	ΔRMSEA
MI for MA/SS										
Config. model (H0)	253.83(202)	0.008	0.962	0.954	0.056					
Scalar model (H1)	267.69(212)	0.006	0.959	0.953	0.056	17.45(10)	0.065	0.003	0.001	0.000
MI for MA/ON										
Config. model (H0)	240.45(202)	0.033	0.952	0.942	0.051					
Scalar model (H1)	252.26(212)	0.030	0.949	0.943	0.05	15.78(10)	0.106	0.003	-0.001	0.001
MI for SS/ON										
Config. model (H0)	214.22(202)	0.264	0.987	0.985	0.036					
Scalar model (H1)	228.94(212)	0.200	0.982	0.980	0.041	20.23(10)	0.027	0.005	0.005	−0.005

Note: H0: Factor loadings and thresholds free across groups, scale factors fixed at one in both groups, and factor means fixed at zero in both groups. H1: Factor loadings and constraints of the thresholds to be equal across groups, scale factors fixed at one in one group and free in the other group, and factor means fixed at zero in one group and free in the other group. CFI: Comparative Fit Index; TLI: Tucker-Lewis Index; RMSEA: Root Mean Square Error Approximation; WLSMV: weighted least square; MI for MA/SS: Measurement of invariance para Moroccans vs. Sub-Saharans; MI for MA/ON: Measurement of invariance para Moroccans vs. Other Nationalities; MI for SS/ON: Measurement of invariance para Sub-Saharans vs. Other Nationalities.

**Table 3:** HLS-EU-Q16 (European Health Literacy Survey Questionnaire) scores.

Variables	*N*	$\bar{X}$ (95% CI)	SD	MEN		WOMEN		(*p*) (CI)
				$\bar{X}$ (95% CI)	SD	$\bar{X}$ (95% CI)	SD	
**HLS-EU-Q16**	205	9.65 (9.03–10.28)	4.51	8.86 (7.78–9.95)	4.97	10.19 (9.46–10.93)	4.10	**(0.046)** (−2.63–0.23)
**Health Care HC-HL**	205	4.09 (3.77–4.41)	2.33	3.81 (3.27–4.35)	2.47	4.27 (3.88–4.67)	2.22	(0.167)
**Prevention DP-HL**	205	2.72 (2.49–2.96)	1.69	2.51 (2.10–2.93)	1.90	2.86 (2.59–3.14)	1.53	(0.165)
**Promotion HP-HL**	205	2.83 (2.65–3.01)	1.30	2.53 (2.21–2.84)	1.44	3.04 (2.83–3.25)	1.17	**(0.007)** (−0.89–0.14)

Note: Although the results of the normality test (Kolmogorov-Smirnov) indicated that the variable followed a non-normal distribution, the parametric student *t* test was used because the sample size was considered to be sufficiently large. CI: Confidence Interval; SD: Standard Deviation; The bold values: significant *p* values.

**Table 4:** Ratings in HLS-EU-Q16 by age group.

Age	Mean Score	SD	X^2^ (*p*)
Less than 35 years	9.05	4.62	6.512 (0.089)
36–50 years	10.74	3.90	
51–65 years	11.08	4.35	
Over 65 years	10.33	5.03	

## Supplementary Materials

The following are available online at https://www.mdpi.com/1660-4601/17/21/8181/s1, Table S1: Final Arabic/French version.
