# Supplementary material for: Psychometric Assessment of the European Health Literacy Survey Questionnaire (HLS-EU-Q16) for Arabic/French-Speaking Migrants in Southern Europe
Source: Int J Environ Res Public Health. 2020 Nov 5;17(21):8181. doi: 10.3390/ijerph17218181 (PMC7663905; doi:10.3390/ijerph17218181)
Supplement: Supplementary file 1 [file ijerph-17-08181-s001.pdf]

Table S1. Final Arabic/ French version

Arabic	French
الحصول على معلومات عن علاجات متعلقة بأمراض تهمك	Trouver des informations sur les traitements liés aux maladies qui sont de votre intérêt.
معرفة أين يمكن الحصول على المساعدة الطبية عندما تكون مريضا (مثل طبيب أو صيدلي أو طبيب نفساني)	Répérer où trouver de l´aide professionnelle quand on est malade
فهم ما يقوله الطبيب	Comprendre ce que dit le medecin
فهم تعليمات الطبيب أو الصيدلي المتعلقة بكيفية تناول الأدوية الموصوفة	Comprendre les indications du médecin ou du farmacien sur la manière de prendre les medicaments inscrits
تقييم متى تكون بحاجة إلى رأي طبي ثاني	Envisager quand peut avoir besoin d´un deuxieme diagnostic d´un autre médecin
استعمال المعلومات المقدمة من طرف الطبيب لإتخاذ قرارات تتعلق بمرضك	Utiliser l´information fournit par le médecin pour soigner la maladie
إتباع تعليمات الطبيب أو الصيدلي	Suivre les inscriptions de son médecin ou de son farmacien.
أو الإجهادبطريقة التعامل مع مشاكل الأمراض العقلية مثل الاكتئاب...	Suivre les renseignements pour trouver la solution aux problèmes mentaux comme le stress ou les troubles depressifs
فهم التحذيرات الصحية المتعلقة ببعض العادات مثل التدخين أو ممارسة الرياضة أو الإفراط في شرب الكحول	Comprendre les avertissements sanitaires liés aux habitudes comme les cigarettes, l excès d´ alcool, le manque des exercice physiques
فهم لماذا تحتاج إلى اختبارات وفحوصات للكشف المبكر عن بعض الأمراض مثل فحص الثدي او قيس نسبة السكر في الدم او قيس الضغط	Comprendre pourquoi on a besoin de faire des analyses de detection précoce de maladies ou des contrôles medicaux
تقييم ما إذا كان بإمكانك الوثوق بالمعلومات المتعلقة بالمخاطر الصحية و التي تظهر بوسائل مثل التلفاز الانترنت او في اي وسيلة اعلامية اخرى	Évaluer la fiabilité de l´information sur les risques contre la santé qui apparaissent sur les médias
أن تقرر كيف تحمي نفسك من الأمراض بفضل المعلومات التي تقدمها وسائل الإعلام (الإنترنت , الجرائد , المجلات ...)	Décide comment se proteger contre les maladies grâce à l´information fournie par les médias
الحصول على أنشطة مفيدة لسلامتك العقلية مثل الاسترخاء او المشي الخ	Trouver des activités appropriées à se tranquilisé mentalement
فهم النصائح الصحية المفيدة التي تقدمها العائلة و الأصدقاء	Comprendre les conseils sur la santé fournie par la famille ou les amis.
فهم المعلومات المقدمة من طرف وسائل الاعلام مثل الانترنت الجرائد المجلات حول كيفية تحسين صحتك	Comprendre l´information fournie par les médias sur l´amélioration de sa santé

تقييم أي من عاداتك اليومية تؤثر على صحتك (التغذية , التدخين , التمارين الرياضية , إلخ.)	Évaluer les habitudes quotidiennes qui aidentent sa santé

Source: Own elaboration
